# Evolution of the human immunodeficiency virus type 2 envelope in the first years of infection is associated with the dynamics of the neutralizing antibody response

**DOI:** 10.1186/1742-4690-10-110

**Published:** 2013-10-24

**Authors:** Cheila Rocha, Rita Calado, Pedro Borrego, José Maria Marcelino, Inês Bártolo, Lino Rosado, Patrícia Cavaco-Silva, Perpétua Gomes, Carlos Família, Alexandre Quintas, Helena Skar, Thomas Leitner, Helena Barroso, Nuno Taveira

**Affiliations:** 1 Unidade dos Retrovírus e Infecções Associadas, Centro de Patogénese Molecular, Faculdade de Farmácia de Lisboa, Lisboa, Portugal; 2 Centro de Investigação Interdisciplinar Egas Moniz (CiiEM), Instituto Superior de Ciências da Saúde Egas Moniz, Monte de Caparica, Portugal; 3 Unidade de Microbiologia Médica, Instituto de Higiene e Medicina Tropical, Universidade Nova de Lisboa, Lisboa, Portugal; 4 Unidade de Imunohematologia, Hospital de Dona Estefânia, Lisboa, Portugal; 5 Laboratório de Biologia Molecular, Serviço de Medicina Transfusional, Centro Hospitalar Lisboa Ocidental–HEM, Lisboa, Portugal; 6 Centro de Malária e Outras Doenças Tropicais, Instituto Superior de Higiene e Medicina Tropical, Lisboa, Portugal; 7 Biology and Biophysics, Los Alamos National Laboratory, Los Alamos, New Mexico, U.S.A

**Keywords:** Vertical HIV-2 infection, Evolution of the neutralizing antibody response, Escape from neutralization, Molecular evolution, Tropism

## Abstract

**Background:**

Differently from HIV-1, HIV-2 disease progression usually takes decades without antiretroviral therapy and the majority of HIV-2 infected individuals survive as elite controllers with normal CD4^+^ T cell counts and low or undetectable plasma viral load. Neutralizing antibodies (Nabs) are thought to play a central role in HIV-2 evolution and pathogenesis. However, the dynamic of the Nab response and resulting HIV-2 escape during acute infection and their impact in HIV-2 evolution and disease progression remain largely unknown. Our objective was to characterize the Nab response and the molecular and phenotypic evolution of HIV-2 in association with Nab escape in the first years of infection in two children infected at birth.

**Results:**

CD4^+^ T cells decreased from about 50% to below 30% in both children in the first five years of infection and the infecting R5 viruses were replaced by X4 viruses within the same period. With antiretroviral therapy, viral load in child 1 decreased to undetectable levels and CD4^+^ T cells recovered to normal levels, which have been sustained at least until the age of 12. In contrast, viral load increased in child 2 and she progressed to AIDS and death at age 9. Beginning in the first year of life, child 1 raised high titers of antibodies that neutralized primary R5 isolates more effectively than X4 isolates, both autologous and heterologous. Child 2 raised a weak X4-specific Nab response that decreased sharply as disease progressed. Rate of evolution, nucleotide and amino acid diversity, and positive selection, were significantly higher in the envelope of child 1 compared to child 2. Rates of R5-to-X4 tropism switch, of V1 and V3 sequence diversification, and of convergence of V3 to a β-hairpin structure were related with rate of escape from the neutralizing antibodies.

**Conclusion:**

Our data suggests that the molecular and phenotypic evolution of the human immunodeficiency virus type 2 envelope are related with the dynamics of the neutralizing antibody response providing further support for a model in which Nabs play an important role in HIV-2 pathogenesis.

## Background

Infection with human immunodeficiency virus type 2 (HIV-2) affects 1-2 million individuals mostly living in West Africa, India and Europe [1,2]. Eight different HIV-2 groups named A through H have been reported but only viruses from groups A and B are known to cause human epidemics [3,4]. Among those, viruses from group A are responsible for the vast majority of HIV-2 infections worldwide.

Even though HIV-1 and HIV-2 are closely related viruses and share a high degree of similarity, infections by these viruses lead to very different immunological and clinical outcomes. HIV-2 infection eventually leads to CD4 depletion, AIDS and death [5-7]. However, differently from HIV-1, HIV-2 disease progression usually takes decades without antiretroviral therapy and the majority of HIV-2 infected individuals survive as elite controllers with normal CD4^+^ T cell counts and low or undetectable plasma viral load [8-16]. Understanding of the factors involved in the effective control of viral replication and disease progression in HIV-2 infected individuals might prove crucial to devise the best strategy to prevent and treat HIV-1.

Enhanced immune control could explain the mild outcome of most HIV-2 infections. Unlike HIV-1 infected patients, most HIV-2 patients in chronic stage produce potent and broad neutralizing antibodies [17-21]. Recent evidence has shown that the viruses isolated from HIV-2 infected patients with advanced disease are characterized by increased resistance to entry inhibitors, including the CCR5-antagonist maraviroc [22] and neutralizing antibodies [23], and by a remarkably high evolutionary rate [24,25]. These results suggest that neutralizing antibodies play a central role in HIV-2 evolution and pathogenesis. However, in contrast to HIV-1, still nothing is known about the neutralizing antibody response and the molecular and phenotypic features of HIV-2 in acute/early infection because HIV-2 patients are usually diagnosed many years after seroconversion.

Most neutralizing epitopes in the HIV-2 envelope glycoprotein complex are located in the surface gp125 glycoprotein. They have been identified in V1, V2, V3, V4 and C5 regions, and in the CD4-binding site [19,21,23,26-29]. These epitopes are well exposed in the envelope complex of CCR5-using isolates that are usually highly sensitive to antibody neutralization [21,27]. However, X4 isolates that emerge in late stage infection in some HIV-2 patients when C2V3C3-specific neutralizing antibodies wane are highly resistant to antibody neutralization [23]. The V3 loop sequence, size and conformation of the X4 isolates are markedly different from those of R5-neutralization sensitive isolates supporting a direct role of this region in escape from neutralization and a direct role of the neutralizing antibodies in shaping the evolution of V3 in progressive HIV-2 infection. The neutralizing domains expressed in the envelope glycoproteins in acute/early infection and the role of the neutralizing antibodies and neutralization escape in shaping the evolution of the HIV-2 envelope in this period remains to be determined.

Perinatal transmission of HIV-2 is a rare event that in Europe has only been documented in Portugal [30-34] and France [35]. Vertical transmission cases constitute a unique opportunity to study the phenotypic and molecular evolution of HIV-2 Env in acute and early infection as well as the role of Nabs in this process. Our objective was to characterize the evolution of the Nab response in two children infected with HIV-2 at birth in association with the molecular and phenotypic evolution of the virus. We show that broad and potent Nabs can be elicited very early after infection and that HIV-2 Env evolves at a very high rate in the first years of infection, this rate being directly associated to the potency of the Nab response. R5-to-X4 tropism change, increased diversity in V1 and V3, and selected changes in V3 conformation were associated with escape from antibody neutralization. The data suggests that the rapid molecular and phenotypic evolution of the HIV-2 envelope in the first years of infection is related with the selective pressure imposed by the neutralizing antibodies.

## Results

### Clinical and virological progression is very fast in the first years of infection

Child 1 infection was diagnosed with HIV-2 infection by PCR and virus isolation in the first month of life in 1998. To confirm the vertical transmission event and characterize the initial infecting virus population, 8 clonal full-length *env* gene sequences were obtained from samples collected in 1998, 2000 and 2003 (in total 24 *env* sequences) and from his mother (mother 1-PTHCC20) in 2000 and 2003 (16 *env* gene sequences). We were unable to obtain 1998 samples from the mother.

Child 2 infection was diagnosed in 1992 at day 39 after birth by PCR and virus isolation, and vertical transmission was confirmed by phylogenetic analysis of partial *env* sequences from the mother and the child [31,32]. Eight new clonal full-length *env* sequences were obtained from samples collected in 1992, 1997 and 2001 (in total 24 *env* sequences).

**Figure 1:**
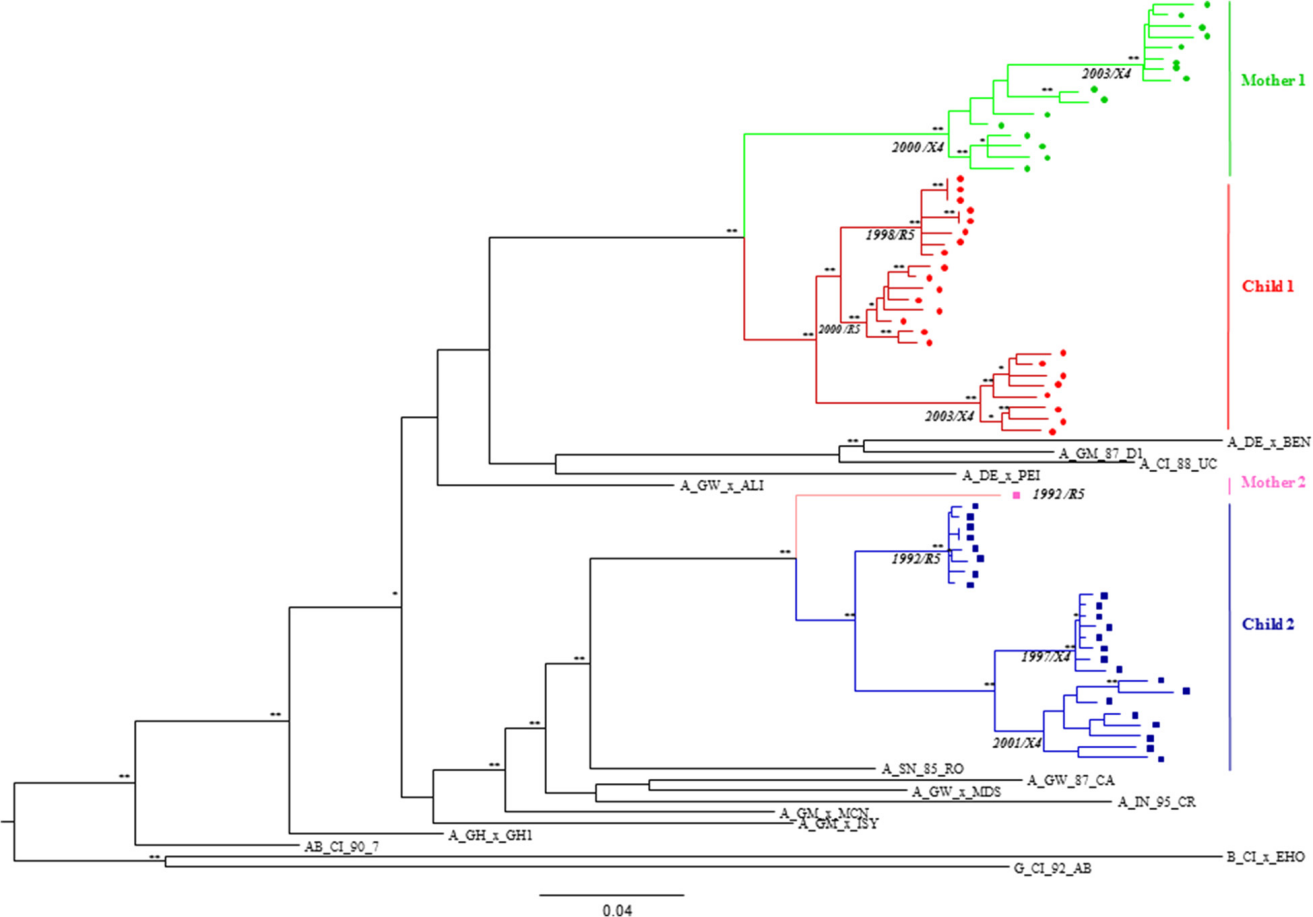
**Evolutionary relationships between mother and child *env *sequences.** A maximum likelihood phylogenetic tree was constructed using alignments of clonal *env* sequences obtained from the children in successive years and from their mothers with reference sequences from HIV-2 groups A, B and G. The bootstrap values supporting the internal branches are shown as follows: * bootstrap >70, ** bootstrap >90. The scale bar represents evolutionary distance in number of substitutions per site. Sequences from child 1 (PTHDECT) are represented by red ircles, with each year of sample collection in orange (1998), red (2000) and dark red (2003); sequences from mother 1 (PTHCC20) are represented by green circles, with each of sample collection in light green (2000) and dark green (2003); sequences from child 2 (PTHDESC) are represented by blue squares with each year of sample collection in light blue (1992), blue (1997) and dark blue (2001); sequence from mother 2 has been published before [32] and is represented by a pink square. The tropism of the viruses is indicated to the right of each cluster.

Phylogenetic analysis showed that all sequences belonged to HIV-2 group A and that mother and child sequences shared a common ancestor, being more closely related to each other than to any other sequences, which confirms the two vertical transmission events (Figure 1). The sequences showed patient-specific clustering, forming sub-clusters corresponding to each year of infection. The sequences from the first sample from both children segregated into one (child 2) or two (child 1) sub-clusters supported by high bootstrap values indicating that one or two virus variants were transmitted from the mothers to the children (Figure 1).

Child 1 was born with normal CD4 percentage (47%) which was sustained until age 3 without ART. The initial infecting virus was CCR5-tropic according to V3 loop sequence analysis of *env* gene clones obtained in 1998 and to phenotypic analysis of virus isolated in 2000 (Table 1 and Figure 1). At age 5, in 2003, CD4 levels decreased to 27%, plasma viral load increased significantly and the virus changed to CXCR4-tropic as determined by phenotypic analysis [22]. Antiretroviral therapy (ART) was initiated at that time leading to a decrease in viral load to undetectable levels and to an increase in CD4^+^ T cells to normal levels. Presently, this child is clinically and immunologically stable and remains asymptomatic.

**Table 1:** Virological and immunological characterization of the patients

**Patient**	**Sampling year**	**Age (Years)**	**Viral load (RNA copies/ml plasma)**	**Viral tropism**^**a**^	**Number of CD4**^**+ **^**T cells/μl (%)**	**Drug regimen**	**Disease stage**^**b**^
Child 1	1998	0.11^c^	<200	R5	5342 (47)	-	N
	1999	1	<200	nd	2992 (50)	-	N
	2000	2	1355	R5	2919 (43)	-	A1
	2001	3	nd	nd	3253 (51)	-	A1
	2003	5	20968	X4	595 (27)	d4T + 3TC + LPV/RTV	B2
	2006	8	<200	nd	1895 (55)	d4T + 3TC + LPV/RTV	A1
	2010	12	<40	nd	1878 (54)	3TC + ABC + LPV/RTV	A1
Child 2	1992	0.07^d^	<200	R5^e^	1670 (52)	AZT	C1
	1997	5	<200	X4	1050 (25)	AZT	C1
	1999	7	13883	nd	127 (15)	AZT	C1
	2001	9	1250	X4	44 (5)	AZT + 3TC	C3 (death)

^a^As determined phenotypically in TZMbl cells [22] or genotypically based on V3 loop sequence patterns [36]; ^b^According to the CDC revised classification system for HIV infection in children; ^c^First blood collection was at day 39 after birth; ^d^First blood collection was at day 27 after birth; ^e^Isolate obtained at birth was syncytium inducing as determined in PBMCs and several cell lines [32]; d4T–stavudine, 3TC–lamivudine, LPV–lopinavir, RTV–ritonavir, ABC–abacavir, AZT–zidovudine; nd–not determined.

Child 2 was born with encephalopathy (CDC clinical stage C1) but with normal CD4^+^ T cell percentage (52%) and undetectable viral load [31,32] (Table 1). The initial infecting virus was CCR5-tropic, as determined by our V3 loop sequence analysis, but induced syncytia formation in peripheral blood mononuclear cells [31,32].

At age 5, CD4 percentage decreased to 25% and the virus changed to CXCR4-tropic, as determined by V3 loop sequence analysis. AZT therapy (1992 up to 1997) and AZT + 3TC therapy (in 2001) did not prevent increase in viral load and further CD4^+^ T cell decline and the child died of AIDS at age 9.

### Potent neutralizing antibodies are produced since the first year of infection especially against R5 isolates

**Figure 2:**
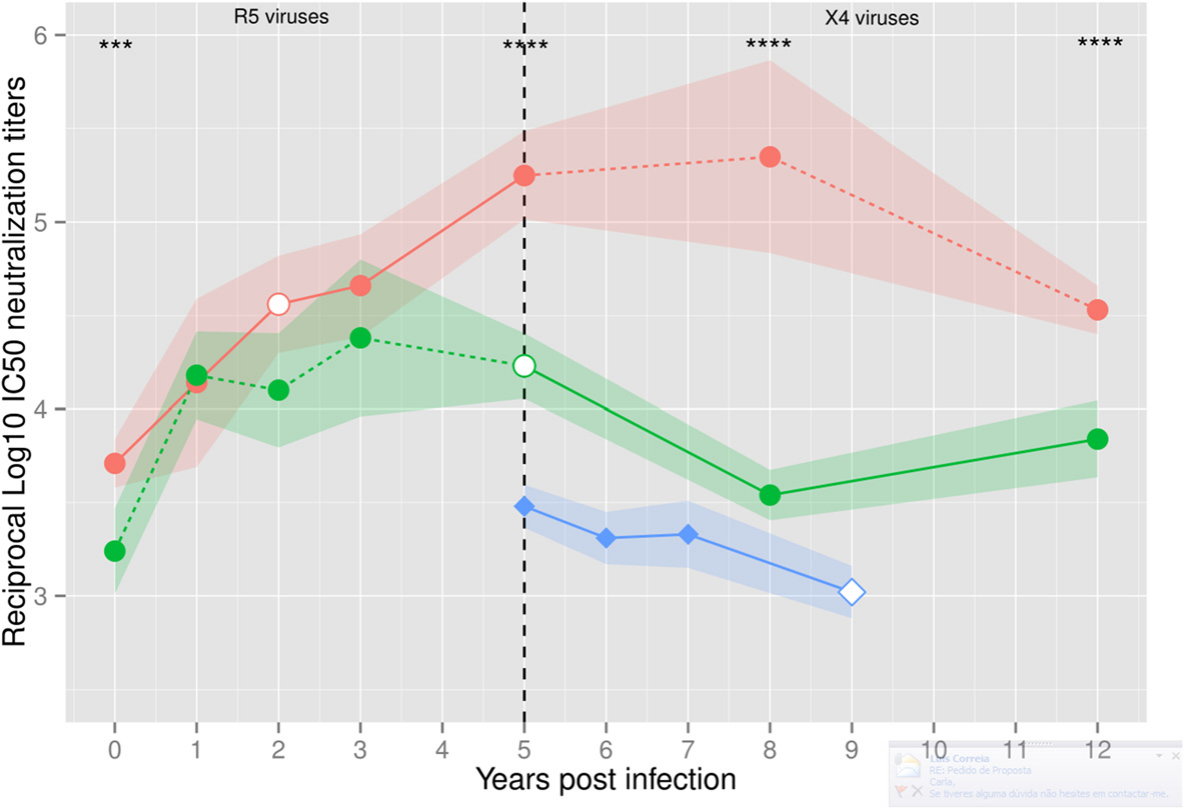
**Evolution of the autologous neutralizing antibody response in the children over the course of infection.** The neutralizing activity present in patients plasma was analyzed against their primary virus isolates using a luciferase reporter gene assay in TZM-bl cells; plasma from child 1 from years 1998, 1999, 2000, 2001, 2003, 2006 and 2010 were tested against autologous viruses from 2000 [CT00 (R5 tropism)–pink circles] and 2003 [CT03 (X4 tropism)–green circles] and plasma from child 2 from years 1997, 1998, 1999 and 2001 were tested against autologous virus from 2001 [SC01 (X4 tropism)–blue diamonds]. Nab titers are presented as median (symbols) and interquartile range (shades) for each serum/virus. Open symbols signal the neutralization of the contemporaneous isolate. In child 1, straight lines represent neutralization of the virus present at that time of infection and dotted lines represent neutralization of virus not present at that time of infection. The F test was used to compare IC50 values obtained for CT00 (R5) and CT03 (X4) isolates. ***P = 0.0008, ****P < 0.0001.

Plasma samples were available from day 27 of birth until the age of 12 for child 1, and from age 5 until age 9 (death) for child 2. Neutralizing activity of these samples was tested against autologous virus isolates obtained at age 2 (R5 isolate, CT00) and at age 5 (X4 isolate, CT03) for child 1, and at age 9 for child 2 (X4 isolate, SC01) (Figure 2). For child 1, the most notorious findings were the steep increase in Nab titers against the initial R5 isolate (CT00) during the first 5 years of infection and the high Nab titers still present at age 12 against this isolate. However, Nabs produced during the initial infection period and during later periods were significantly less potent against the X4 isolate that emerged in 2003 at age 5 (CT03) [median (range) of reciprocal log_10_ IC50 neutralization titers against CT00 and CT03 were 4.6 (3.7-5.4) and 4.1 (3.2-4.4), respectively, P = 0.0472, Mann–Whitney test]. This difference in susceptibility to neutralization of the two isolates was particularly evident from age 5 onwards when the X4 virus emerged in the child. The close associations between rates of Nab escape and R5-to-X4 phenotypic switching suggests that phenotype transition in this infant is directly related with the Nab pressure.

**Figure 3:**
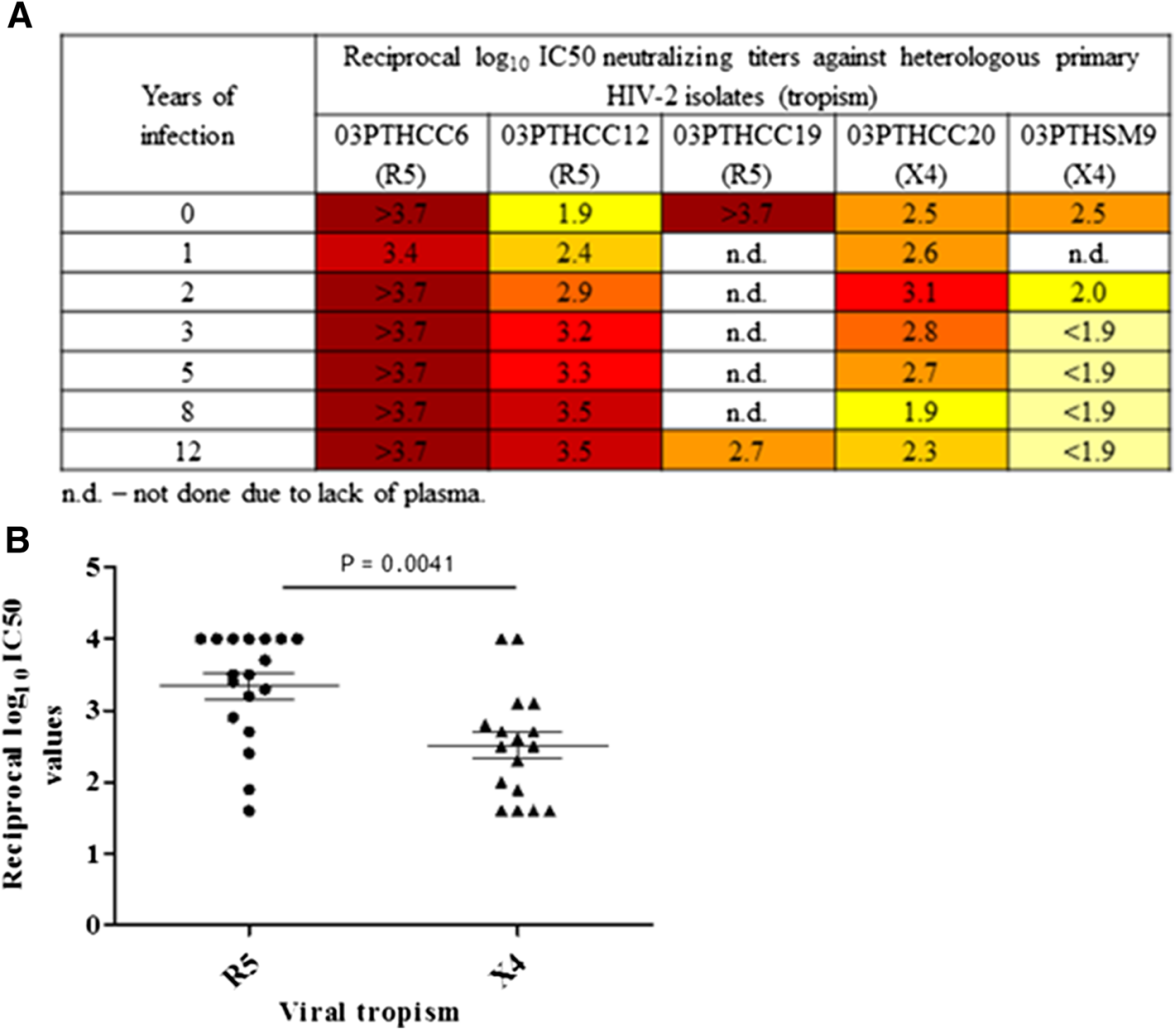
**Neutralizing antibody response against heterologous primary isolates in child 1 over the course of infection. A)** A heat map of the reciprocal log-transformed IC50 value of each plasma sample from child 1 (left) against a panel of five heterologous primary virus isolates with respective tropism (top) is shown. The reciprocal log_10_ IC50 value is colour-coded. The darkest colour indicates that neutralization above 50% was still detected with the highest plasma dilution tested (1/5120). The lightest colour indicates that there was no detectable neutralization above 50% with the lowest plasma dilution tested (1/40). n.d.–not done (due to lack of plasma); **B)** Dot-plot graphic showing the mean and standard deviation of the reciprocal log_10_ IC50 values obtained against R5 and X4 isolates indicated in A. Mann–Whitney U test was used to compare the median log_10_ reciprocal IC50 values.

Notably, child 1 also produced neutralizing antibodies that potently neutralized several heterologous primary HIV-2 isolates. Again, the heterologous Nabs were significantly more effective against R5 strains than against X4 strains [median (range) of reciprocal log_10_ IC50 neutralization titers against R5 and X4 isolates were 3.5 (1.6-4.0) and 2.5 (1.6-4.0), respectively, P = 0.0041] (Figure 3).

In child 2 we could only analyse the evolution of Nab response against the autologous X4-isolate (SC01) from age 5 onwards. Comparing Nab response at age 5 in both patients (the only age-matched data point), we found that it was significantly weaker in child 2 than in child 1 [median (range) of reciprocal log_10_ IC50 neutralization titer of child 2 against SC01 was 3.5 (3.4-3.6), and those of child 1 against autologous CT00 and CT03 isolates were 5.3 (5.0-5.5) and 4.2 (4.0-4.4), respectively, P < 0.001] (Figure 2). Moreover, in contrast to child 1, Nab titer decreased steadily with infection time as viral load increased and disease progressed to AIDS and death at age 9 (Figure 2 and Table 1). Considering all time points together, average Nab titers were lower than those of child 1 against the age-matched X4 isolate CT03 [median (range) of reciprocal log_10_ IC50 neutralization titer against isolates SC01 and CT03 were 3.3 (3.0-3.5) and 4.1 (3.2-4.4), respectively, P = 0.057] and against the R5 isolate CT00 [median (range) of reciprocal log_10_ IC50 neutralization titer against isolates SC01 and CT00 were 3.3 (3.0-3.5) and 4.6 (3.7-5.4), respectively, P = 0.0106].

HIV-negative plasmas failed to neutralize HIV-2 strains and HIV-2 plasmas failed to neutralize HIV-1SG3.1 or viruses pseudotyped with VSV envelope indicating the absence of nonspecific inhibitory activities in these samples.

Overall, the results obtained with child 1 demonstrate that potent neutralizing antibodies (autologous and heterologous) can be elicited very rapidly after HIV-2 vertical infection. Nabs are highly effective against the transmitted R5 isolates but seem to rapidly select for X4 isolates that escape neutralization. In the absence of effective antiretroviral therapy, as was the case of child 2, increased replication of the Nab-resistant X4 isolates likely contributed to rapid CD4^+^ T cell depletion and progression to AIDS.

### HIV-2 Env evolution in acute/early and late infection

At birth, nucleotide diversity in child 1 was twice that of child 2 in *env* and five times in C2V3C3 region (Table 2). At age 5, nucleotide diversity increased 2-fold in *env* and C2V3C3 in child 1 while in child 2 it only increased in C2V3C3 (2-fold) leading to an even higher difference in *env* and C3V3C3 diversity (3-and 6-fold, respectively). Interestingly, in child 2, diversity increased significantly from age 5 onward, both in *env* (4-fold when compared to diversity at age 5) and C2V3C3 (11-fold) exceeding that of child 1 at age 5. However, in contrast to the first years of infection, most substitutions occurring in this period were of a synonymous nature as indicated by the sharp decrease in the ω value both in *env* and C2V3C3.

**Table 2:** **Nucleotide diversity and divergence rates in the *env *** **gene and C2V3C3 region**

**Patient**	**Sampling year**	**Age (years)**	**ω**^**a**^		**Nucleotide diversity**^**b **^**(SD)**		**Evolutionary rate**^**c **^**(95% HPD)**	
			**env**	**C2V3C3**	**env**	**C2-V3-C3**	**env**	**C2V3C3**
Child 1	1998	0.11	0.96	1.52	0.013 (0.0061)	0.014 (0.0063)	0.0141 (0.0075, 0.0211)	0.0142 (0.0082, 0.0208)
	2000	2	0.88	5.78	0.027 (0.0047)	0.015 (0.0074)		
	2003	5	0.65	0.50	0.027 (0.0057)	0.031 (0.0118)		
Child 2	1992	0.07	1.15	0.38	0.007 (0.0022)	0.003 (0.0026)	0.0073 (0.0036, 0.0115)	0.0105 (0.0053, 0.0174)
	1997	5	0.99	2.60	0.008 (0.0025)	0.005 (0.0026)		
	2001	9	0.66	0.62	0.035 (0.0122)	0.055 (0.0157)		

^a^Ratio of nonsynonymous to synonymous substitution rates; ^b^Within-patient genetic distances and standard deviation (SD) as determined by averaging pairwise tree distances over all the sequences obtained for each patient at each time point; ^c^Nucleotide substitutions per site per year (HPD, highest posterior density).

The evolutionary rate of *env* was significantly higher in child 1 than in child 2 (0.0141 *vs* 0.0073 substitutions/site/year, posterior probability (PP) value <5%) (Table 2). When focusing on the C2V3C3 region, the evolutionary rates were not significantly different (0.0142 *vs* 0.0105 substitutions/site/year, PP=20%). There was a trend towards positive selection in child 1 (non-synonymous rate: *env*, 0.0143 and V3, 0.0152; synonymous rate: *env*, 0.0137 and V3, 0.0124 substitutions/site/year) and purifying selection in child 2 (non-synonymous rate: *env*, 0.0069 and V3, 0.0092; synonymous rate: *env*, 0.0082 and V3, 0.0132 substitutions/site/year) in both *env* and V3.

**Figure 4:**
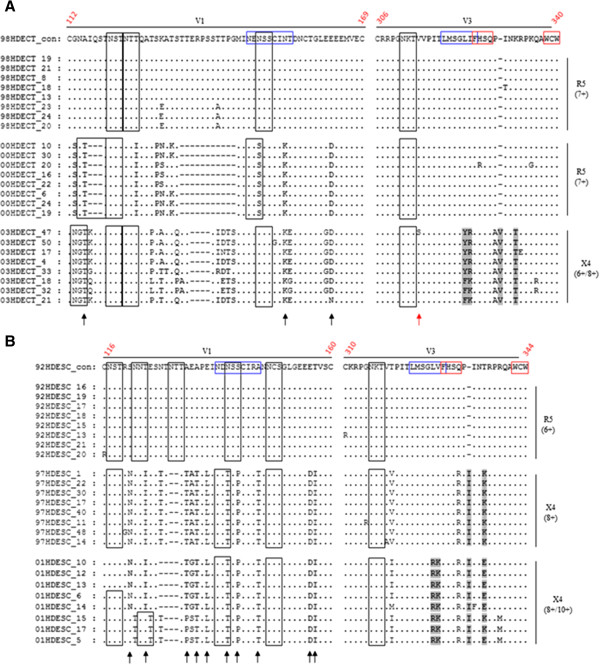
**Evolution of V1 and V3 regions.** Clonal V1 and V3 amino acid sequences obtained over the course of infection from child 1 **(panel A)** and child 2 **(panel B)** were aligned against consensus sequences from the initial infecting isolate. Dots in the alignments indicate sequence identity to the consensus; dashes indicate deletions. Potential N-linked glycans (occurring at NXT/S) are represented in open boxes. Amino acids highlighted in light grey are involved in tropism change [36]. Blue boxes in the consensus sequence represent published linear neutralizing epitopes in V1 [28] and V3 [26]; red boxes in the consensus sequence represent a conformational epitope in V3 [26]. Viral tropism is indicated at the right of the alignment as determined phenotypically or genotypically based on V3 loop sequence patterns. Black arrows signal fixed mutations.

We also analysed the evolution of amino acid diversity, as determined by the sum of Shannon’s entropy, in variable regions of gp125 which contains most of the neutralizing domains [19,21,26,27]. At birth, amino acid diversity was higher in child 1 than in child 2 (Table 3). At age 5, amino acid diversity increased significantly only in V1 and V3 in both patients, this being much more pronounced in child 1. In child 2, from age 5 to age 9 (death), amino acid diversity increased in V1 (9.3-fold), V3 (2.1-fold) and V4 (1.6-fold), though never reaching the level of diversity observed in child 1 at age 5. Amino acid changes observed after the first year in V1 and V3 are shown in Figure 4. In V1 there was no clear pattern of change except for the 2-4 amino acids deletion detected at year 5 in both patients. This deletion was maintained along the full course of infection in child 2. In child 1, three mutations occurring at age 3 were fixed (were kept in year 5) and 4 mutations reversed back to the original residue suggesting that these changes affected viral fitness; in child 2, ten mutations were fixed over the course of infection and there were no reversions suggesting that the mutations did not reduce the fitness of the virus or that compensatory mutations occurred in other regions. Three of the fixed mutations in child 2 were located in a previously described neutralizing epitope [26]. Likewise, in child 1 two mutations of a potentially disruptive nature emerged in this neutralizing epitope (N to K and T to E/G).

**Table 3:** Evolution of amino acid diversity in variable Env regions in the first five years of infection

**Patient**	**Variable regions**	**Sum of entropy**		**Fold increase**
		**year of birth**	**year 5**	
child 1	V1	1.324	8.657	6.5
	V2	0	1.885	na
	V3	0.754	3.614	4.8
	V4	0.939	0.662	na
	V5	0	3.402	na
	V1-V5	3.017	18.22	6.0
child 2	V1	0.377	0.754	2.0
	V2	1.131	0.377	na
	V3	0.377	1.131	3.0
	V4	0	1.316	na
	V5	0.377	0	na
	V1-V5	2.262	3.578	1.6

na- not applicable.

In V3, mutations occurred almost exclusively within previously described neutralizing epitopes [26], and at residues 18, 19 and 27 that have been associated with R5 and X4 tropism [36-38]. One amino acid insertion occurred in the same position in both children and involved a hydrophobic residue (V in child 1; I in child 2). This type of insertion has also been associated to R5-to-X4 tropism switch [36,37].

Env adaptation to Nab pressure is usually associated with positive selection of specific amino acids that might be located in neutralizing domains [39]. At year 5 of infection there were 10 positively selected sites in Env of child 1 (seven in gp125) (Table 4). Most sites (6 out of 10) were located in confirmed neutralizing domains (V2, V3 and C5 in gp125 and MPER in gp36) [27]. In contrast, positively selected sites were absent in child 2 at year 5 of infection and there were only 2 selected sites in the final year of infection. These results reveal a much better adaptation to Nab pressure in child 1 compared to child 2.

**Table 4:** Positive selective pressure on the Env glycoproteins in both children over the course of infection

**Env glycoprotein**	**Codons under selective pressure (location)**^**1**^					
	**Child 1**			**Child 2**		
	**Year of birth**	**Year 2**	**Year 5**	**Year of birth**	**Year 5**	**Year 9**
gp125	none	5, 7 (in SP)	178 (V2), 255, 259 (C2), 320 (V3), 459 (V5), 467, 471 (C5)	none	none	395 (C3)
gp36	none	none	552 (HR1), 672, 673 (MPER)	none	none	562 (HR1)

^1^Codons identified as being significantly (P <0.05) under selective pressure are indicated; SP, signal peptide; V2, variable region 2; C2, conserved region 2; C3, conserved region 3; V3, variable region 3; V5, variable region 5; C5, conserved region 5; HR1, helical region 1; MPER, membrane proximal external region.

In all, these results show that HIV-2 *env* can evolve and diversify very rapidly in the first years of infection. The positive correlation between the rate of Env evolution, in terms of nucleotide divergence from the initial virus, nucleotide diversity, amino acid diversity, and positive selection, and the rate of Nab response and escape indicates that Nabs likely have a major impact on HIV-2 Env evolution in the first years of infection.

### Tropism and susceptibility to antibody neutralization are closely associated with V3 structure

**Figure 5:**
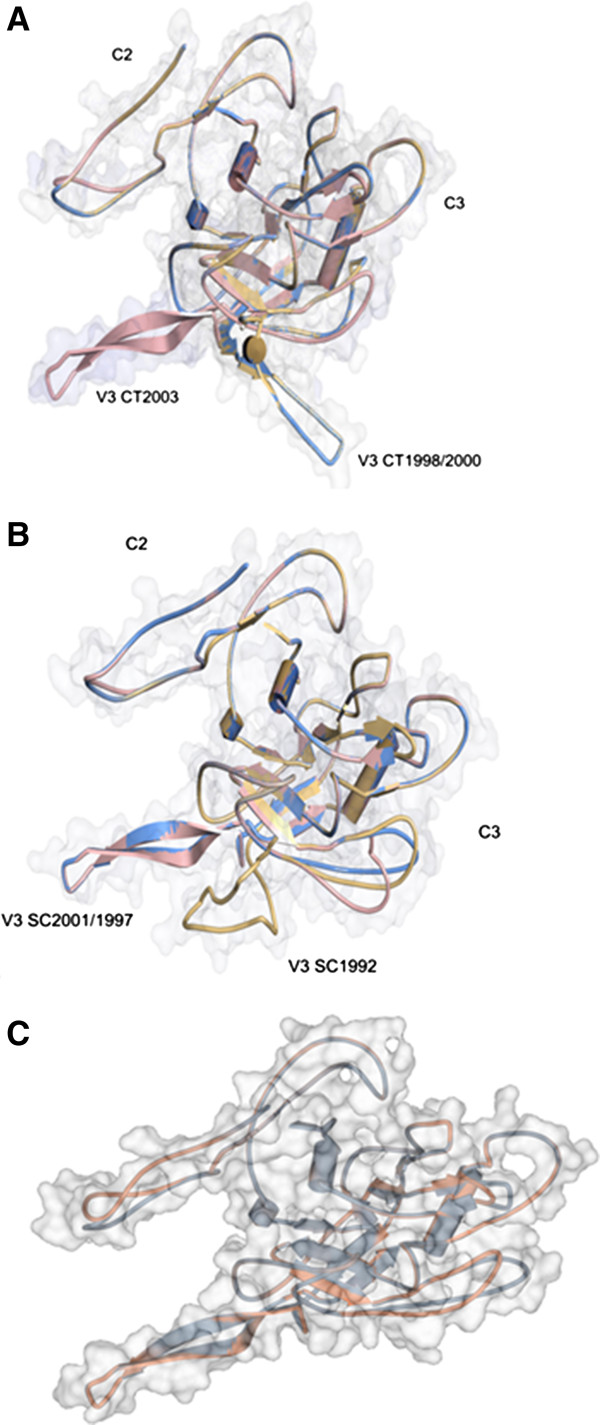
**Evolution of the structure of C2-V3-C3 envelope region.** Three-dimensional structures of C2-V3-C3 amino acid sequences from child 1 and 2 were generated by homology modelling using the three-dimensional structure of an unliganded SIV gp120 envelope glycoprotein as template. **A)** Superimposition of the structures of C2-V3-C3 of child 1 in 1998 (yellow), 2000 (blue) and 2003 (pale red); **B)** Superimposition of the structures of C2-V3-C3 of child 2 in 1992 (yellow), 1997 (blue) and 2001 (pale red). V3 loop and C2 and C3 stretches are indicated in the figures. **C)** Three-dimensional structures of C2-V3-C3 from child 1 (CT) in 2003 (blue) and child 2 (SC) in1997 (red). At this time both viruses were X4 and showed resistance to Nabs.

In long-term HIV-2 infected individuals the envelope V3 region adopts a significantly different structure in Nab-resistant isolates as compared to Nab-sensitive isolates, supporting a direct role of V3 conformation in the different susceptibility of these viruses to antibody neutralization [23]. To gain some insight into the structural evolution of the V3 region in the first years of HIV-2 infection and try to relate it to tropism and susceptibility to antibody neutralization, model structures of C2-V3-C3 regions from both children were generated by homology modelling using the three-dimensional structure of an unliganded SIV gp120 envelope glycoprotein as template. Remarkably, the V3 loop, which was characterized by a high content of irregular secondary structure in the first year of infection, converged to an similar β-hairpin structure at year five of infection in both infants and remained in this conformation until the last year of infection in child 2 (Figure 5 and Additional file 1: Table S1). The rate of acquisition of the β-hairpin conformation fully correlated with the rate of R5-to-X4 tropism transition and with the rate of escape from antibody neutralization.

## Discussion

There is limited knowledge on the natural history of HIV-2 infection and on the molecular and phenotypic evolutionary dynamics of HIV-2 because no study has investigated the full course of infection from the time of seroconversion. The current study is the first characterization of the Nab response and molecular and phenotypic evolution of HIV-2 followed from acute infection to late stage infection. Our studies were based on two children infected by vertical transmission and spanned the first 12 years of infection in one case and the complete infection period in the other (9 years). We show that a potent Nab response is raised very early after infection and that the rate and pattern of molecular and phenotypic evolution of the HIV-2 Env are closely associated to the rate of Nab escape.

Child 2 was born severely ill despite the normal CD4^+^ T cell percentage [31,32] whereas child 1 was born asymptomatic and with normal CD4 levels. Despite the contrasting clinical conditions at birth, major CD4 depletion and disease progression occurred in both children in the first 5 years of infection. This fast disease course is typical of HIV-1 infected children in the absence of antiretroviral treatment [40] but is highly unusual in HIV-2 infected individuals [10,34,41,42]. Both patients were infected with R5 strains but transitions to X4 tropism occurred rapidly, being detected after only 5 years of infection. This is the first time that a full R5-to-X4 tropism switch is observed in HIV-2 infected patients and it was unexpected to find it in paediatric patients. Like in some adult HIV-2 patients with advanced disease [23,37,43], the emergence of X4 viruses in our patients was associated with high viral load, marked CD4 depletion and disease progression. Hence, the rapid disease course in the two infants may have been determined by the early emergence of X4 isolates.

At birth, HIV-2 nucleotide diversity in child 1 was 2-fold higher than in child 2 both in *env* and C2V3C3. Nucleotide diversity in child 1 was also 2-fold higher than in HIV-1 infected children in the first weeks after birth [44-46] and in HIV-1 adult patients during seroconversion [47]. Envelope diversity also increased more significantly, both at the nucleotide and amino acid levels, in child 1 than in child 2 in the first 5 years of infection. Consistently, the evolutionary rate of the *env* gene in child 1 was almost two times higher than child 2 and similar to that found in chronic HIV-2 infected patients under ART (0.0102 substitutions/site/year) [24]. These results reveal a remarkably high rate of molecular evolution of the HIV-2 envelope in child 1 during the first 5 years of infection and a moderate rate of evolution in child 2. The evolutionary rate in child 2 was similar to that in HIV-1 patients who, when untreated, have a disease progression generally similar to that of child 2. Thus, the higher rate in child 1 is consistent with the better immune control.

Previously, we have shown that production of gp36-specific and gp125-specific antibodies occurred during the first year of age in child 1 and that, at age 2, levels of binding antibodies to these glycoproteins were already similar to those found in HIV-2 infected patients in the chronic stage of disease [16]. In child 2, although gp36-specific antibodies were produced to near normal levels, there was a remarkably weak production of gp125-specific binding antibodies. Consistently, in this work we found that child 1 produced a much stronger Nab response than child 2. In child 1 autologous neutralizing antibodies appeared within the first year of infection, increased over time to levels that were similar to chronically infected patients [18,19,21], and were sustained until at least the age of 12. Moreover, child 1 also produced a potent Nab response against several heterologous virus isolates. As for child 2, the autologous Nab titer was significantly lower compared to child 1 at age 5 and decreased continuously to very low titers following the rapid decline of CD4 cells and progression to AIDS and death at age 9. Similar findings have been reported in late stage disease adult patients in whom Nab titers decrease in direct association with CD4^+^ T cell depletion [23]. Overall, these results suggest that a potent Nab response during the acute/early phase of infection might contribute to control HIV-2 disease progression. Further investigation of the relationship between Nab response and disease progression in the acute and early stages of HIV-2 infection of adult patients is required as this might provide new insights into the benign course of most HIV-2 infections.

The differences in Nab response between the two children also correlate well with the magnitude and rate of envelope evolution in the infants which suggest a close association between the neutralizing antibody response and the evolution of the HIV-2 envelope in these patients. Several lines of evidence further suggest that escape from Nab response is a major determinant of the evolution of the HIV-2 envelope in these infants, especially in child 1. First, complete replacement of virus quasispecies was noted in phylogenetic analysis of *env* sequences produced at the different time points which is compatible with a situation of ongoing viral escape from antibody neutralization [48]. Second, amino acid diversity increased significantly with infection time, especially in V1 and V3 which are major neutralizing domains in HIV-2. This is a major HIV escape mechanism as a single polymorphism can alter epitope sequence and/or conformation and reduce recognition and/or binding affinity by neutralizing antibodies [39,48-52]. Third, increase in dN/dS ratio and positive selection in the envelope were closely related to rate of Nab escape in child 1 [39,51]. Finally, the similar gain of secondary structure in V3 in both patients fully correlated with the rate of escape from antibody neutralization. This has been recently associated to HIV-2 resistance to antibody neutralization in chronic HIV-2 infected patients [23].

Nabs were significantly more potent against R5 isolates than against X4 isolates (autologous and heterologous) confirming the inherent resistance of X4 viruses to antibody neutralization [23,53]. More importantly, increase in Nab resistance in child 1 preceded the emergence of X4 variant suggesting that tropism switch may have been driven by the neutralizing antibodies. Given the immunodominance of the V3 region in HIV-2 infected patients [54], the location of two of the three amino acid residues that are associated to R5 and X4 tropism (positions 18 and 19) [36-38] within the first neutralizing epitope in V3 [26] and the major difference in V3 conformation of R5 and X4 strains [23], the close association between HIV-2 susceptibility to antibody neutralization and tropism seen in these infants is not surprising.

The main limitations of this study are the small number of patients and the inexistence of viral isolates from all time points in both children. However, worldwide it has been impossible to find individuals acutely infected with HIV-2. Moreover, due to the low or absent viral load it is often impossible to isolate virus from HIV-2 infected patients. Notwithstanding these limitations, we believe that our results are a major contribution to our understanding of the natural history of HIV-2 infection and of the role of the immune system in controlling and shaping HIV-2 evolution.

## Conclusions

In conclusion, we show that a potent Nab response is elicited very early after HIV-2 infection and that the HIV-2 envelope evolves at a high rate in the first years of infection, this rate being directly correlated to the potency of the Nab response. R5-to-X4 tropism switch, increased nucleotide and amino acid diversity in V1 and V3, and convergence of V3 to a β-hairpin structure were closely associated with escape from the Nab response suggesting that Nabs have a major impact on the rapid molecular and phenotypic evolution of the viral envelope in acute and early in HIV-2 infection. Our studies provide further support to a model of HIV-2 pathogenesis in which Nabs play a central role.

## Methods

### Study subjects and ethics

Two children infected by vertical transmission were studied. Blood samples were collected from child 1 (patient PT.HDE.CT), 39 days after birth in 1998, in 1999, 2000, 2001, 2003, 2006 and 2010, and from child 2 (patient PT.HDE.SC), 27 days after birth in 1992, in 1997 and 2001. Clinical and immunological characteristics of the patients are shown in Table 1. Child 1 started ART (stavudine + lamivudine + lopinavir/ritonavir) in November 2003. Presently, the child is taking lamivudine + abacavir + lopinavir/ritonavir; his viral load is undetectable and he is clinically and immunologically stable. Child 2 started ART with zidovudine immediately after birth and lamivudine was added in 2001. In 2001, viral load increased slightly and CD4+ T cells decreased sharply leading to the child’s death. Ethical approval was obtained from the Ethics Committee of Hospital Curry Cabral and written informed consent was obtained from the children's parents before entry into the study.

### HIV-2 env gene PCR amplification, cloning and sequencing

Chromossomal DNA was extracted from infected PBMC’s using Wizard Genomic DNA Purification Kit (Promega) according to the manufacturer recommendations. A 2600 bp fragment encompassing the entire *env* gene was amplified by nested Polymerase Chain Reaction (PCR) using the Expand Long Template PCR Systemkit (Roche) and newly designed primers (Additional file 2: Table S2). The PCR protocol consisted of denaturation at 95°C for 5 min, 35 amplification cycles of 15 sec at 94°C, 30 sec at 59°C and 3 min at 68°C with 5 sec increments and a final elongation step at 68°C for 30 min. 5 μl of PCR product was used as the template for nested PCR. The amplification profile of the nested PCR was identical to the first PCR, except for annealing temperature and extension time (61°C and 2 min respectively). PCR amplicons were purified with a JETQUICK Gel Extraction Spin Kit (Genomed). For each sample, PCR products were cloned into the pcDNA3.1/V5-His-TOPO vector (Invitrogen), using the TOPO TA Expression Kit (Invitrogen) according to the manufacturer’s instructions. At least eight clones from each patient/year were sequenced using the BigDye Terminator V3.1 Cycle sequencing Kit (Applied Biosystems); sequencing primers are displayed in Additional file 2: Table S2. Sequencing was performed on an ABI 3100–Avant Genetic Analyzer (Applied Biosystems).

### Sequence analysis

Clustal × 2.1 [55] software was used to construct alignments of HIV-2 *env* sequences. Reference HIV-2 sequences were obtained from the Los Alamos National Laboratory HIV sequence database [56]. Maximum likelihood phylogenetic analyses were performed using the best-fit model of molecular evolution estimated in PAUP by Modeltest using likelihood ratio tests [57]. The chosen model was GTR + I + G. Tree searches were conducted in PAUP using nearest-neighbour interchange (NNI) and tree-bisection plus reconnection (TBR) heuristic search strategies [58], and bootstrap resampling with 1000 replicates [59]. The genetic distances between sequences were calculated by averaging pairwise tree distances using all sequences obtained for each patient at each time point, as previously described [60]. Putative recombinants were identified using the Phi-statistic [61] available in SplitsTree version 4.10 [62] by performing 10 randomized reductions of putative recombinants. Putative recombinant sequences were removed before doing the evolutionary rate analyses. These were: 00PTHDECT_9, 00PTHDECT_16, 00PTHDECT_22, 00PTHDECT_6, 00PTHDECT_24, 00PTHDECT_19, 00PTHDECT_8, 00PTHDECT_12, 03PTHDECT_17, 03PTHDECT_33, 03PTHDECT_21, 01PTHDESC_13, 01PTHDESC_6 and 01PTHDESC_14.

Selective pressure on the HIV-2 Env was examined with the DATAMONKEY web-server [63], after removing all positions containing gaps and missing data from the dataset. All estimations were performed using the MG94 codon substitution model [64] crossed with the nucleotide substitution model GTR, previously selected with Modeltest (see above). The single-likelihood ancestor counting (SLAC) method was used to infer the ratio of nonsynonymous to synonymous nucleotide substitutions (dN/dS) averaged over all codon positions of the alignment. To identify individual codons under selective pressure, site-specific dN/dS rates were estimated by the relaxed-effects likelihood (REL) method, with a cut-off value for the Bayes factor of 50 [65].

The Bayesian program BEAST was used to estimate the nucleotide evolutionary rates [66]. The SRD06 model [67] of substitution was used and two different clock models were used, relaxed lognormal and strict clock. A constant parametric demographic model as well as the non-parametric Skyline plot with 3 groups was tested. The MCMC chains were chosen so that the effective sample size for all parameters exceeded 300 and convergence was assessed by inspecting the traces in the program Tracer [68]. Appropriate demographic and molecular clock models were chosen by examining the marginal posterior distributions of relevant parameters.

Potential N-linked glycosylation sites were identified using the N-Glycosite software [69] and the entropy at each position in protein alignment was measured with Shannon’s entropy [70], both available at the Los Alamos National Laboratory HIV sequence database [56].

### Virus isolation and tropism characterization

Primary virus isolates were obtained from both patients using the co-cultivation method as described previously [32]. Viral tropism (CCR5 and/or CXCR4 usage) was determined in TZM-bl cells in the presence of CCR5 or CXCR4 antagonists as described previously [22]. Tropism was also determined genetically using the V3 loop clonal sequences and the algorithm described by Visseux*et al*[36] which is based in the sequence, size and charge of the V3 loop.

### Neutralization assay

The neutralizing activity present in patients serum was analyzed against autologous and heterologous primary virus isolates using a luciferase reporter gene assay in TZM-bl cells, as described previously [2,53,71]. Briefly, the cells [10,000 cells in 100 μl of complete growth medium (GM) that consists of DMEM supplemented with 10% heat-inactivated fetal bovine serum (FBS)], were added to each well of 96-well flat-bottom culture plates (Nunc) and allowed to adhere overnight. One hundred μl of each virus (corresponding to 200 TCID50) were incubated for 1 h at 37°C with 2-fold serial dilutions of heat-inactivated patients sera in a total volume of 200 μl of GM containing DEAE-Dextran (20 μg/ml). The lowest serum dilution used in the assays was 1:80. Forty-eight hours later, plates were analyzed for luciferase activity on a luminometer (TECAN) using the One-Glow Luciferase Assay System (Promega, Madison, WI). Medium only control wells were measured as background, and virus-only control wells were included as 100% infection. Neutralization titers were expressed as the reciprocal of the plasma dilution that inhibited virus infection by 50% (IC50). IC50 was estimated by the sigmoidal dose–response (variable slope) equation in Prism version 5.0 [72]. Nonspecific inhibition was assessed by testing all HIV-2 isolates against HIV-negative plasma and all plasma s∆env as backbone).

### Structural models

Structural models of the C2-V3-C3 domain in gp125 were produced with SWISS-MODEL homology modelling server in automated mode, using PDB file 2BF1 (SIV) as template [73,74]. Accelrys Discovery Studio 2.1 (Accelrys Inc., San Diego, USA, 2008) was used to produce three dimensional images of the obtained models and perform the secondary structure analysis of the V3 loop.

### Statistical analysis

Statistical analysis was performed with GraphPad Prism 5.0 [72] with a level of significance of 5%. F test was used to compare best fit values of IC50 slopes obtained with CT00 and CT03 isolates from child 1. Non parametric Mann Whitney test was used to compare autologous Nab responses (mean IC50s) between child 1 and child 2. To compare evolutionary rates we computed the posterior probability (PP) that one rate exceeded the other and the probability was determined numerically by randomly sampling from the empirical posterior distributions [24]. Kruskal-Wallis test was used to compare mean Shannon’s entropies between variable Env regions of both patients.

### GenBank accession numbers

Full-length envelope sequences generated in this study are available from GenBank under the following accession numbers: GU983917-GU983940 and JX219591-JX219614.

## Competing interests

The authors declare that they have no competing interests.

## Authors’ contributions

NT and TL designed the study; CR, HS, TL, AQ and NT analysed the data and wrote the paper; CR performed the experiments; CR, HS and TL, performed the evolutionary analysis;,CR, RC, PB, JMM, IB and HB provided analytical reagents and nucleotide sequences; CR, PB and NT performed the statistical analysis; CF and AQ performed the structural analysis; PG performed the viral load assays; LR and PCS contributed clinical data from the patients. The final text was read and approved for submission by all authors.

## Supplementary Material

Additional file 1: Table S1 Percentage of major secondary structure motifs present in the V3 loop of HIV-2 isolates obtained from child 1 and 2. Click here for file

Additional file 2: Table S2 PCR and sequencing primers for the HIV-2 env gene. ^a^Outer PCR primer; ^b^Inner PCR primer; ^c^Sequencing primer. Click here for file
