# Supplementary material for: Evolution of the human immunodeficiency virus type 2 envelope in the first years of infection is associated with the dynamics of the neutralizing antibody response
Source: Retrovirology. 2013 Oct 24;10:110. doi: 10.1186/1742-4690-10-110 (PMC4016255; doi:10.1186/1742-4690-10-110)
Supplement: Additional file 1: Table S1 — Percentage of major secondary structure motifs present in the V3 loop of HIV-2 isolates obtained from child 1 and 2. [file 1742-4690-10-110-S1.docx]

**Table S2 -** Percentage of major secondary structure motifs present in the V3 loop of HIV-2 isolates obtained from child 1 and 2

| Secondary structure | Child 1 (CT) | | | Child 2 (SC) | | |
| --- | --- | --- | --- | --- | --- | --- |
|  | 1998 | 2000 | 2003 | 1992 | 1997 | 2001 |
|  | (R5 virus) | (R5 virus) | (X4 virus) | (R5 virus) | (X4 virus) | (X4 virus) |
| α-helix | 11.8 % | 11.8 % | 0.0 % | 0.0 % | 0.0 % | 0.0 % |
| β-sheet | 41.2 % | 41.2 % | 71.4 % | 5.9 % | 71.4 % | 71.4 % |
| Turn | 11.8 % | 11.8 % | 11.4 % | 20.6 % | 11.4 % | 11.4 % |
| Random coil | 35.2 % | 35.2 % | 17.2 % | 73.5 % | 17.2% | 17.2 % |
