# Supplementary material for: Evolution of the human immunodeficiency virus type 2 envelope in the first years of infection is associated with the dynamics of the neutralizing antibody response
Source: Retrovirology. 2013 Oct 24;10:110. doi: 10.1186/1742-4690-10-110 (PMC4016255; doi:10.1186/1742-4690-10-110)
Supplement: Additional file 2: Table S2 — PCR and sequencing primers for the HIV-2 env gene. aOuter PCR primer; bInner PCR primer; cSequencing primer. [file 1742-4690-10-110-S2.docx]

**Table S1 -** PCR and sequencing primers for the HIV-2 *env* gene

| **Primer** | **Position** | **Sequence 5’ – 3’** |
| --- | --- | --- |
| CR1^a^ | 5927 – 5946 | aggaaacag(c,t)gg(a,c)gaagaga |
| CR2^a^ | 9391 – 9369 | tctacatcatccatattttg(c,t)tg |
| CR3^b^ | 6673 – 6692 | ctcat(c,t)cgtcttctgcatca |
| CR4^b^ | 9286 – 9268 | tcacaggagggcgatttct |
| CRSEQ2^c^ | 7363 – 7345 | atcccaatagtgctt(a,g)tca |
| CRSEQ3 ^c^ | 7313 – 7334 | cattgcaacacatcagtcatca |
| CRSEQ4 ^c^ | 7918 - 7898 | ccaattgaggaaccaagtcat |
| CRSEQ5 ^c^ | 7859 – 7879 | atgtggactaactgcagagga |
| CRSEQ6 ^c^ | 8360 – 8344 | gctgttgctgttgctgc |
| CRSEQ7 ^c^ | 8344 – 8360 | gcagcaacagcaacagc |
| CRSEQ8 ^c^ | 8835 – 8817 | gagaaaacaggcctatagc |
| CRSEQ9 ^c^ | 8817 – 8835 | gctataggcctgttttctc |
| CRSEQ10 ^c^ | 7159 – 7173 | agacaattgcacagg |
| CRSEQ11 ^c^ | 7424 – 7410 | tggtatcattgcatc |

^a^Outer PCR primer; ^b^Inner PCR primer; ^c^Sequencing primer
